# Performance of Artificial Intelligence Chatbots on Glaucoma Questions Adapted From Patient Brochures

**DOI:** 10.7759/cureus.56766

**Published:** 2024-03-23

**Authors:** Goutham R Yalla, Nicholas Hyman, Lauren E Hock, Qiang Zhang, Aakriti G Shukla, Natasha N Kolomeyer

**Affiliations:** 1 Department of Ophthalmology, Sidney Kimmel Medical College, Thomas Jefferson University, Philadelphia, USA; 2 Glaucoma Research Center, Wills Eye Hospital, Philadelphia, USA; 3 Department of Ophthalmology, Vagelos College of Physicians and Surgeons, Columbia University, New York, USA; 4 Department of Ophthalmology, Glaucoma Division, Columbia University Irving Medical Center, New York, USA; 5 Biostatistics Consulting Core, Vickie and Jack Farber Vision Research Center, Wills Eye Hospital, Philadelphia, USA

**Keywords:** bard, bing, chatgpt, patient education, chatbot, artificial intelligence, glaucoma

## Abstract

Introduction

With the potential for artificial intelligence (AI) chatbots to serve as the primary source of glaucoma information to patients, it is essential to characterize the information that chatbots provide such that providers can tailor discussions, anticipate patient concerns, and identify misleading information. Therefore, the purpose of this study was to evaluate glaucoma information from AI chatbots, including ChatGPT-4, Bard, and Bing, by analyzing response accuracy, comprehensiveness, readability, word count, and character count in comparison to each other and glaucoma-related American Academy of Ophthalmology (AAO) patient materials.

Methods

Section headers from AAO glaucoma-related patient education brochures were adapted into question form and asked five times to each AI chatbot (ChatGPT-4, Bard, and Bing). Two sets of responses from each chatbot were used to evaluate the accuracy of AI chatbot responses and AAO brochure information, and the comprehensiveness of AI chatbot responses compared to the AAO brochure information, scored 1-5 by three independent glaucoma-trained ophthalmologists. Readability (assessed with Flesch-Kincaid Grade Level (FKGL), corresponding to the United States school grade levels), word count, and character count were determined for all chatbot responses and AAO brochure sections.

Results

Accuracy scores for AAO, ChatGPT, Bing, and Bard were 4.84, 4.26, 4.53, and 3.53, respectively. On direct comparison, AAO was more accurate than ChatGPT (p=0.002), and Bard was the least accurate (Bard versus AAO, p<0.001; Bard versus ChatGPT, p<0.002; Bard versus Bing, p=0.001). ChatGPT had the most comprehensive responses (ChatGPT versus Bing, p<0.001; ChatGPT versus Bard p=0.008), with comprehensiveness scores for ChatGPT, Bing, and Bard at 3.32, 2.16, and 2.79, respectively. AAO information and Bard responses were at the most accessible readability levels (AAO versus ChatGPT, AAO versus Bing, Bard versus ChatGPT, Bard versus Bing, all p<0.0001), with readability levels for AAO, ChatGPT, Bing, and Bard at 8.11, 13.01, 11.73, and 7.90, respectively. Bing responses had the lowest word and character count.

Conclusion

AI chatbot responses varied in accuracy, comprehensiveness, and readability. With accuracy scores and comprehensiveness below that of AAO brochures and elevated readability levels, AI chatbots require improvements to be a more useful supplementary source of glaucoma information for patients. Physicians must be aware of these limitations such that patients are asked about existing knowledge and questions and are then provided with clarifying and comprehensive information.

## Introduction

Glaucoma is the leading cause of irreversible blindness globally, with an estimated 76 million individuals affected and over 110 million individuals estimated to be affected by 2040 [1]. Given the chronic course of the disease, visual prognosis is dependent on long-term adherence to treatment regimens, which is shaped by patient education [2]. While ophthalmologists are conventionally a source of patient education, curious patients can be inundated by online medical information in the form of thousands of websites and social media platforms. An estimated 43% of glaucoma patients utilize the Internet for medical information, with varied evaluations of the quality, benefits, and harms of information from online resources [3-7].

Most recently, the advent of artificial intelligence (AI)-based chatbots, such as ChatGPT, has presented a new avenue for patients to access medical information quickly. Unlike websites or social media, AI chatbots present information to patients by answering questions in a direct and interactive form. The knowledge patients obtain in response to their questions could craft their understanding and concerns of glaucoma, shaping patient adherence and physician-patient encounters.

With the potential for AI chatbots to serve as the primary source of medical information to patients, it is essential to characterize the information that AI chatbots provide such that providers can tailor discussions, anticipate patient concerns, and identify misleading information. The utility of ChatGPT in providing ophthalmic information has been evaluated for examination questions, retinal diseases, and keratoconjunctivitis but has yet to be assessed for glaucoma-related questions [8-11]. Further, studies on AI chatbots have been limited to ChatGPT and have not considered alternative available AI chatbots that have the potential to reach millions of patients. Our study aims to evaluate glaucoma information from available AI chatbots, including ChatGPT-4 by OpenAI, Bard by Google, and Bing by Microsoft, by analyzing response accuracy, comprehensiveness, readability, word count, and character count in comparison to each other and glaucoma-related American Academy of Ophthalmology (AAO) patient materials.

## Materials and methods

The most current AAO glaucoma-related patient education brochures (2022) were collected, including “Glaucoma,” “Laser Iridotomy,” “Laser Trabeculoplasty,” “Trabeculectomy,” and “Glaucoma Drainage Implant.” Each section header was adapted into question form, suitable for AI chatbot input (Table 1). On April 18, 2023, all 19 questions were asked to three AI chatbots: ChatGPT-4 by OpenAI (March 23 version), Bing by Microsoft, and Bard by Google (Bard Experiment). Each question was asked five times to each AI chatbot to generate five sets of responses from each chatbot. Each question was entered into a new “conversation,” such that no prior responses were present in the chat history. Default ChatGPT settings were used. The conversation style for the Bing AI chatbot was set to the default setting of “more balanced.” Bard presented an option to view alternative response “drafts” to each question; however, the default response to each question was utilized.

**Table 1:** AAO Brochure Titles and Questions Used for AI Chatbot Input AAO: American Academy of Ophthalmology, AI: artificial intelligence

AAO Brochure Title	Question
Glaucoma	
	What is glaucoma?
	What causes glaucoma?
	What are the types of glaucoma?
	Who is at risk for glaucoma?
	How is glaucoma diagnosed?
	How is glaucoma treated?
	What is my role in glaucoma treatment?
Laser Iridotomy	
	What is laser iridotomy?
	How is laser iridotomy performed?
	What are the risks of laser iridotomy?
Laser Trabeculoplasty	
	What is laser trabeculoplasty?
	How is laser trabeculoplasty performed?
	What are the risks of laser trabeculoplasty?
Trabeculectomy	
	What is a trabeculectomy?
	How is trabeculectomy performed?
	What are the risks of trabeculectomy?
Glaucoma Drainage Implant	
	What is a glaucoma drainage implant?
	How is glaucoma drainage implant surgery performed?
	What are the risks of glaucoma drainage implant surgery?

Accuracy analysis and comprehensiveness of AI chatbot responses

Three glaucoma fellowship-trained ophthalmologists (NNK, AGS, and LEH) evaluated the accuracy of the AAO brochure information and AI chatbot responses, as well as the comprehensiveness of AI chatbot responses compared to the information in the AAO brochures. Each ophthalmologist evaluated two sets of responses from each AI chatbot. The six chatbot response sets were blinded and randomized; an investigator (GRY) used a random number generator to assign a number to each response set, unique for each ophthalmologist (NNK, AGS, and LEH). Accuracy was assessed according to the following scale: 1 = agreement with <25% of the information, 2 = agreement with 25%-50% of the information, 3 = agreement with 51%-75% of the information, 4 = agreement with 76%-99% of the information, and 5 = agreement with 100% of the information. Comprehensiveness was assessed according to the following scale: 1 = much less than the AAO brochure section, 2 = slightly less than the AAO brochure section, 3 = similar to the AAO brochure section, 4 = slightly more than the AAO brochure section, and 5 = much more than the AAO brochure section.

Readability, word count, and character count analysis

The readability, word count, and character count of each AAO brochure section and each AI chatbot response from all collected responses (five sets of responses from each chatbot) were determined using a Microsoft Word document (version 2306) (Microsoft Corp., Redmond, WA). Readability was assessed using the Flesch-Kincaid Grade Level (FKGL), a reading level corresponding to the United States school grades calculated from the number of syllables, words, and sentences in each response.

Source analysis of Bing AI chatbot responses

The Bing chatbot provides references for each of its responses. Sources from Bing chatbot responses were collected and categorized into the following categories: research organizations/foundations, academic institutions, private practices, independent websites, commercial entities, and peer-reviewed papers. Sources were collected from all five sets of responses to each question, given their variations.

Attending comments

The glaucoma specialist graders (NNK, AGS, and LEH) commented on AI chatbot responses, providing specific critiques of responses otherwise not captured from alternative analyses. Comments were organized into three categories: incorrect, phrasing concerns, and lack of comprehensive information.

Statistical analysis

Mean and standard deviation (SD) were calculated for accuracy scores, comprehensiveness scores, readability, word count, and character count, with values grouped by resource (AAO, ChatGPT, Bing, and Bard). Accuracy and comprehensiveness scores were compared between the resources via Friedman tests with the Wilcoxon signed-rank test for between-group comparisons. Readability, word count, and character count were compared between the resources via repeated measures analysis of variance (ANOVA) tests with Bonferroni post hoc analysis for between-group comparisons. A two-sided p-value of <0.05 was considered statistically significant for all tests apart from the multiple comparisons (Wilcoxon signed-rank test, p<0.05/n: accuracy - p<0.0083, comprehensiveness - p<0.0166). Sources of Bing responses were evaluated by determining the number of sources for each Bing response, the number of source variants between the five Bing responses to each question (variant defined as a unique set of sources), and the percent breakdown of sources by category. All analyses were done using SAS (version 9.4).

## Results

AI chatbot analysis

Mean (standard deviation) accuracy scores, comprehensiveness scores, readability scores, word count, and character count for AAO, ChatGPT, Bing, and Bard were determined, with direct comparison between groups (Table 2). Most notably on direct comparison, AAO was more accurate than ChatGPT (p=0.002) and Bard (p<0.001), ChatGPT was more accurate than Bard (p=0.002), and Bing was more accurate than Bard (p=0.001). Although the accuracy score of Bing fell in between AAO and ChatGPT, differences between Bing and these two groups were not statistically significant (Table 2). On direct comparison, ChatGPT responses were the most comprehensive of the AI chatbots, AAO information and Bard responses were at the most accessible readability levels, and Bing responses had the lowest word and character count (Table 2). The distribution of average accuracy scores for AAO and AI chatbot responses was determined, with the greatest percentage of scores between 4 and 5 seen in AAO, followed by Bing, ChatGPT, and lastly Bard (Table 3).

**Table 2:** AAO Brochure and AI Chatbot Response Evaluation Accuracy and comprehensiveness are scored from 1 to 5. Comprehensiveness is scored relative to AAO information. Standard deviations are presented in parentheses. Significant p-values are bolded (threshold for significance: p<0.0083 for direct comparisons of accuracy scores, p<0.0166 for direct comparison of comprehensiveness scores, and p<0.05 for remaining tests). M: mean, SD: standard deviation, AAO: American Academy of Ophthalmology, AI: artificial intelligence

	AAO	ChatGPT	Bing	Bard	p-value overall	p-value AAO versus ChatGPT	p-value AAO versus Bing	p-value AAO versus Bard	p-value ChatGPT versus Bing	p-value ChatGPT versus Bard	p-value Bing versus Bard
Accuracy, M (SD)	4.84 (0.38)	4.26 (0.56)	4.53 (0.51)	3.53 (0.77)	<0.001	0.002	0.034	<0.001	0.096	0.002	0.001
Comprehensiveness, M (SD)	-	3.32 (0.75)	2.16 (1.12)	2.79 (1.03)	<0.001	-	-	-	<0.001	0.008	0.001
Readability, M (SD)	8.11 (1.46)	13.01 (0.65)	11.73 (2.08)	7.90 (0.65)	<0.0001	<0.0001	<0.0001	1	0.0234	<0.0001	<0.0001
Word count, M (SD)	174.53 (129.87)	222.26 (29.17)	100.77 (38.33)	247.36 (59.47)	<0.0001	0.3292	0.0213	0.0238	<0.0001	1	<0.0001
Character count, M (SD)	864.58 (641.31)	1151.15 (114.18)	528.26 (212.09)	1178.88 (270.79)	<0.0001	0.1197	0.0401	0.0659	<0.0001	1	<0.0001

**Table 3:** Distribution of Average Accuracy Scores Between AAO Brochures and AI Chatbots AAO: American Academy of Ophthalmology, AI: artificial intelligence

Average accuracy score range	AAO	ChatGPT	Bing	Bard
≥4 to ≤5	100% (19/19)	63.2% (12/19)	89.5% (17/19)	26.3% (5/19)
≥3 to <4	0% (0/19)	36.8% (7/19)	10.5% (2/19)	68.4% (13/19)
≥2 to <3	0% (0/19)	0% (0/19)	0% (0/19)	5.3% (1/19)
≥1 to <2	0% (0/19)	0% (0/19)	0% (0/19)	0% (0/19)

Source analysis of Bing AI chatbot responses

The mean (standard deviation) source per response was 4.7 (0.7), with a range of 4-6. For each question asked to Bing, the mean (standard deviation) number of source variants between the five responses was 2.6 (0.7), with a range of 2-4. Sources were categorized, with the greatest percentage of sources from independent websites (40%), followed by research organizations (36.2%), academic institutions (12.9%), private practices (4.7%), commercial entities (3.7%), and peer-reviewed papers (2.6%).

Attending comments

Representative glaucoma specialist grader comments from all AI chatbots were included according to their themes: incorrect, phrasing concerns, and lack of comprehensive information (Table 4).

**Table 4:** Representative Commentary of AI Chatbot Responses AI: artificial intelligence, IOP: intraocular pressure

Theme	Chatbot response context	Sample comments from reviewers
Incorrect	Laser iridotomy is typically performed as an outpatient procedure and is generally well-tolerated. The procedure is usually quick and painless.	This procedure is not painless.
	The risk of bleeding can be reduced by taking blood thinners before the surgery	The opposite is true.
	Laser trabeculoplasty helps lower the pressure inside the eye by creating a new drainage pathway.	Works on natural drains and does not create new pathways.
	(In regard to trabeculectomy) The surgeon uses a special contact lens to keep the eye still.	This is a step of laser trabeculoplasty and not trabeculectomy.
	(In regard to trabeculectomy) A small piece of tissue is removed from the trabecular meshwork, a network of tiny channels that drain fluid from the eye. A new drainage channel is created in the sclera. The flap of the conjunctiva is replaced. The incision is closed with stitches.	Multiple scenarios when responses confuse surgical anatomy and sequence of steps.
	(In regard to iridotomy) The hole is usually located in the upper part of the iris, under the upper eyelid.	An example of a statement that may have been true in the past, but not currently, as other locations are possible besides superior.
Phrasing concerns	As with any surgery, there are some risks associated with glaucoma drainage implant surgery. These risks include: … 5. glaucoma recurrence.	Cure is not a term we use with glaucoma, as optic nerve damage caused by glaucoma cannot be cured at this time. Therefore, if glaucoma is never cured, it cannot recur.
	Here are some things you can expect after glaucoma drainage implant surgery: … 4. you will need to use eye drops to help keep your eye pressure low.	It is not certain that patients will need to use eye drops; some patients are able to get off their pressure-lowering eye drops. They will need eye drops to decrease the risk of inflammation and infection immediately after surgery.
	Glaucoma is caused by high pressure inside the eye.	This is not always correct. This is sometimes a misconception about glaucoma.
	Angle closure glaucoma: this type of glaucoma occurs when the iris bulges forward, blocking the eye’s drainage system and leading to a sudden increase in eye pressure.	This talks about only acute angle closure glaucoma. Most angle closure glaucoma is chronic and does not have these acute events.
	The exact cause of glaucoma is not fully understood, but it is thought to be caused by a combination of factors, including: 1. high IOP, 2. family history of glaucoma, 3. age, 4. race, 5. certain medical conditions, such as diabetes or hypertension, and 6. eye trauma.	Most of these are not causes; they are risk factors for glaucoma.
Lack of comprehensive information	As with any surgery, there are some risks associated with glaucoma drainage implant surgery. These risks include: 1. infection, 2. bleeding, 3. damage to the eye, 4. cataract, 5. glaucoma recurrence, and 6. the need for additional surgery.	No mention of risks including diplopia, blurry vision, ptosis, high or low IOP, and loss of vision.
	Congenital glaucoma is a type of glaucoma that is present at birth. It is caused by an abnormality in the drainage angle of the eye. Secondary glaucoma is a type of glaucoma that is caused by another eye condition, such as an eye tumor, an infection, or an injury.	There are other types of glaucoma also that are not included, such as pigmentary, pseudoexfoliation, uveitic, and neovascular.

## Discussion

The interactive format of AI chatbots such as ChatGPT presents an accessible source of medical information to millions of inquisitive patients globally. With an estimated 43% of glaucoma patients utilizing the Internet for medical information, it is essential for physicians to understand both the information AI chatbots provide and how it is provided, such that physicians are best equipped to guide patient education and improve patient adherence [2,3]. In this study, available AI chatbots, including ChatGPT-4 by OpenAI, Bard by Google, and Bing by Microsoft, were evaluated for their accuracy, comprehensiveness, readability, word count, and character count.

AAO brochure glaucoma information was the most accurate, although the accuracy scores of Bing and ChatGPT closely followed, suggesting their utility to patients. Bard was significantly less accurate than alternative AI chatbots, limiting its ability to provide glaucoma information. While any amount of inaccuracy introduces the risk of patient harm, the performance of AI chatbots is more appropriately evaluated in the context of alternative information resources available to patients, which have been websites rather than gold-standard materials such as AAO brochures. A prior investigation analyzing the top 15 websites that resulted when “glaucoma” was searched via Google demonstrated that only 26% of websites were graded in the “75%-100% accurate” category [4]. In the present study, 63% of ChatGPT responses, 90% of Bing responses, and 26% of Bard responses were graded as “76%-100% accurate.” These results suggest that in comparison to individual website searches, Bing and ChatGPT provide patients with more accurate glaucoma information. However, the information queried to assess accuracy varied across these studies, and therefore, a direct comparison cannot be made.

Literature establishing the accuracy of ChatGPT in providing answers to ophthalmic questions is varied. Rasmussen et al. rated 56% of ChatGPT responses to questions on vernal keratoconjunctivitis as having no inaccuracies or minor non-harmful inaccuracies [10]. Potapenko et al. demonstrated greater accuracy, with 71% of retinal disease question responses graded to have no inaccuracies or minor non-harmful inaccuracies [11]. Momenaei et al. evaluated responses for surgical treatments of retinal diseases and found that answers were graded as appropriate in 93% of 264 total responses, utilizing a scale of “appropriate,” “inappropriate,” or “incomplete” [8]. These variations in accuracy are partially explained by the variations in scoring systems and their granularity; however, it raises the possibility that ChatGPT, and perhaps other AI chatbots, are better versed at answering questions on certain topics.

While the accuracy of responses is most critical when evaluating patient education resources, as inaccuracies can lead to patient harm and misunderstandings, responses that are insufficient do not equip patients with the necessary information. Instead, patients may feel a false sense of assurance that they have completely understood their question and may not opt to speak to their physician about their concern. Although the accuracy of ChatGPT has been evaluated in select domains, the comprehensiveness of the information provided by AI chatbots has yet to be objectively characterized in any field, including ophthalmology. Compared with the gold standard AAO brochures, ChatGPT was the most comprehensive in its responses, followed by Bard and Bing. Despite the comprehensiveness score of ChatGPT indicating superiority over the AAO brochures, AAO brochures contain several critical concepts that patients may not have independently considered. Although self-evident, since AI chatbots only answer questions that are asked, there is potential for fewer questions to be asked, resulting in less information being obtained as opposed to a standard patient information document, such as the AAO brochures.

Although accurate and comprehensive information is essential to best educate patients, if information is not understood, it is of limited use. AAO and Bard responses were found to be at the eighth-grade level, Bing responses were at a 12th-grade level, and ChatGPT responses were provided at a first-year collegiate level. The readability of glaucoma-related ChatGPT responses is consistent with an investigation of the readability of retina-related ChatGPT responses, which was found to be at a second-year collegiate level, emphasizing the comprehension difficulties associated with this chatbot [8]. Alternative institutional and online patient educational glaucoma materials have shown to be written at a 10th- to 12th-grade level, with online glaucoma information to be presented at a 9th- to 11th-grade level [4,12-16]. Given that the American Medical Association recommends less than a seventh-grade readability level for educational materials, AI chatbot responses, apart from Bard (which contains inaccuracies as previously mentioned), were written at levels that would be challenging to comprehend for most patients [17]. If information from ChatGPT and Bing cannot be understood by those with low health literacy, differences in patient comprehension between literacy groups may increase with the popularization of these AI chatbots. Low health literacy has been associated with a greater number of medications and reduced outpatient follow-up visits for glaucoma patients [18]. These vulnerable groups are especially in need of high-quality, comprehensible patient education. This may be accomplished through physicians or vetted materials such as the AAO brochures or by improving chatbots to adhere to the recommended reading level guidelines.

The length of AI chatbot responses may influence their user-friendliness. Briefer responses, measured by word and character counts, may be more straightforward for patients to interpret and understand. Bing responses were found to be significantly briefer than alternative chatbots and the AAO responses; however, its brevity parallels its reduced comprehensiveness scores, suggesting that rather than being succinct, Bing responses lacked sufficient information. Despite being longer, the word and character counts of ChatGPT, Bard, and AAO materials are approximately the length of a paragraph and are therefore of reasonable length.

Bing provided citations for each of its responses, a feature unique to this chatbot that adds credibility as sources can be reviewed. Although independent websites were the most common type of source, over half were from research organizations, academic institutions, or peer-reviewed papers. This distribution of sources is similar to the study of the top 15 websites when “glaucoma” was searched via Google, suggesting that Bing may synthesize the most popular website information without selecting for more reliable sources [4].

In addition to the objective evaluation of accuracy and comprehensiveness, a subjective assessment of AI chatbot responses by glaucoma specialists revealed certain patterns in the incorrect responses and illustrated how AI chatbots can potentially cause harm to patients. The lack of comprehensive information, such as the exclusion of certain risks of glaucoma drainage implant surgery, may misguide patients. Phrasing concerns can perpetuate misconceptions, such as a response stating that “glaucoma is caused by high pressure in the eye.” Incorrect procedure details such as “the surgeon uses a special contact lens to keep the eye still” when describing trabeculectomy, while not actionable information to patients, may need clarification during physician visits. The most harmful statements from AI chatbots include false information that can lead to adverse patient outcomes. The response “the risk of bleeding can be reduced by taking blood thinners before surgery” is harmful if the patient does not have an explicit conversation about this topic with their physician, and it is difficult for physicians to know what false recommendations have been made by AI chatbots. The presence of harmful inaccuracies in ChatGPT responses has been established by several studies in both ophthalmology and other medical fields [10,11,19]. To minimize this potential danger, physicians should elicit patient questions, provide detailed patient instructions, and encourage patients to talk to physicians before making any medical decisions.

Limitations of the study should be considered when interpreting the results. The questions evaluated by the study were derived from AAO brochures, which may not mirror patients’ most common glaucoma-related questions. When assessing the comprehensiveness of the AI chatbots, they were done so in relation to the AAO brochures, therefore assuming the AAO brochures contained the most comprehensive information. Also, both accuracy and comprehensiveness were evaluated with subjective scoring scales. However, as the comprehensiveness was directly compared to the AAO information and the accuracy scores were based on numerical percentages as opposed to an “accurate or inaccurate” system, this limitation was minimized. Given that the AI chatbots are continuously updated and, in some cases, new information is made available to them, a limitation of this study is that all responses were generated on a single day. Notably, study results may have been different if settings other than the default were used for AI chatbots. Future studies may opt to examine responses over longer periods of time to evaluate for consistency and improvements in parameters over time.

## Conclusions

In conclusion, our study evaluated the strengths and limitations of multiple AI chatbots, including ChatGPT, Bing, and Bard, in answering glaucoma-related questions. Physicians must be aware of these limitations such that patients are asked about existing knowledge and questions and are then provided with clarifying and comprehensive information. AI developers can improve glaucoma-related chatbot responses by improving readability and reducing inaccuracies with the use of more accurate online sources and glaucoma specialists. With improvements, AI chatbots may be a useful supplementary source of glaucoma information to enhance patient education in the future.

## References

[1] Tham YC, Li X, Wong TY, Quigley HA, Aung T, Cheng CY (2014). Global prevalence of glaucoma and projections of glaucoma burden through 2040: a systematic review and meta-analysis. Ophthalmology.

[2] Quaranta L, Novella A, Tettamanti M, Pasina L, Weinreb RN, Nobili A (2023). Adherence and persistence to medical therapy in glaucoma: an overview. Ophthalmol Ther.

[3] Stagg BC, Gupta D, Ehrlich JR, Newman-Casey PA, Stein JD, Kawamoto K, Hess R (2021). The use of eHealth practices by United States patients with self-reported glaucoma. Ophthalmol Glaucoma.

[4] Jia JS, Shukla AG, Lee D, Razeghinejad R, Myers JS, Kolomeyer NN (2022). What glaucoma patients are reading on the Internet: a systematic analysis of online glaucoma content. Ophthalmol Glaucoma.

[5] Tıskaoğlu NS, Seyyar SA (2022). #Ophthalmology: popular ophthalmology hashtags as an educational source for ophthalmologists, an Instagram study. Indian J Ophthalmol.

[6] Dave AD, Zhu D (2022). Ophthalmology inquiries on Reddit: what should physicians know?. Clin Ophthalmol.

[7] Oydanich M, Shah Y, Shah K, Khouri AS (2022). An analysis of the quality, reliability, and popularity of YouTube videos on glaucoma. Ophthalmol Glaucoma.

[8] Momenaei B, Wakabayashi T, Shahlaee A (2023). Appropriateness and readability of ChatGPT-4-generated responses for surgical treatment of retinal diseases. Ophthalmol Retina.

[9] Antaki F, Touma S, Milad D, El-Khoury J, Duval R (2023). Evaluating the performance of ChatGPT in Ophthalmology: an analysis of its successes and shortcomings. Ophthalmol Sci.

[10] Rasmussen ML, Larsen AC, Subhi Y, Potapenko I (2023). Artificial intelligence-based ChatGPT chatbot responses for patient and parent questions on vernal keratoconjunctivitis. Graefes Arch Clin Exp Ophthalmol.

[11] Potapenko I, Boberg-Ans LC, Stormly Hansen M, Klefter ON, van Dijk EH, Subhi Y (2023). Artificial intelligence-based chatbot patient information on common retinal diseases using ChatGPT. Acta Ophthalmol.

[12] Huang G, Fang CH, Agarwal N, Bhagat N, Eloy JA, Langer PD (2015). Assessment of online patient education materials from major ophthalmologic associations. JAMA Ophthalmol.

[13] Williams AM, Muir KW, Rosdahl JA (2016). Readability of patient education materials in ophthalmology: a single-institution study and systematic review. BMC Ophthalmol.

[14] Cheng BT, Kim AB, Tanna AP (2022). Readability of online patient education materials for glaucoma. J Glaucoma.

[15] Crabtree L, Lee E (2022). Assessment of the readability and quality of online patient education materials for the medical treatment of open-angle glaucoma. BMJ Open Ophthalmol.

[16] Cohen SA, Fisher AC, Pershing S (2023). Analysis of the readability and accountability of online patient education materials related to glaucoma diagnosis and treatment. Clin Ophthalmol.

[17] Weiss BD (2007). Health literacy and patient safety: help patients understand.

[18] Cheng BT, Tanna AP (2023). Association of health literacy and health care utilization among glaucoma patients. J Glaucoma.

[19] Sarraju A, Bruemmer D, Van Iterson E, Cho L, Rodriguez F, Laffin L (2023). Appropriateness of cardiovascular disease prevention recommendations obtained from a popular online chat-based artificial intelligence model. JAMA.

